# The Jacob2 Lectin of the *Entamoeba histolytica* Cyst Wall Binds Chitin and Is Polymorphic

**DOI:** 10.1371/journal.pntd.0000750

**Published:** 2010-07-20

**Authors:** Sudip K. Ghosh, Katrina L. Van Dellen, Anirban Chatterjee, Tuli Dey, Rashidul Haque, Phillips W. Robbins, John Samuelson

**Affiliations:** 1 Department of Molecular and Cell Biology, Boston University Goldman School of Dental Medicine, Boston, Massachusetts, United States of America; 2 Department of Biotechnology, Indian Institute of Technology Kharagpur, Kharagpur, India; 3 International Centre for Diarrhoeal Disease Research, Bangladesh (ICDDR,B), Dhaka, Bangladesh; New York University School of Medicine, United States of America

## Abstract

**Background:**

The infectious and diagnostic form of *Entamoeba histolytica* (Eh), cause of amebic dysentery and liver abscess, is the quadranucleate cyst. The cyst wall of *Entamoeba invadens* (Ei), a model for Eh, is composed of chitin fibrils and three sets of chitin-binding lectins that cross-link chitin fibrils (multivalent Jacob lectins), self-aggregate (Jessie lectins), and remodel chitin (chitinase). The goal here was to determine how well the Ei model applies to *Entamoeba* cysts from humans.

**Methods/Results:**

An Eh Jacob lectin (EhJacob2) has three predicted chitin-binding domains surrounding a large, Ser-rich spacer. Recombinant EhJacob2 made in transfected Eh trophozoites binds to particulate chitin. Sequences of PCR products using primers flanking the highly polymorphic spacer of EhJacob2 may be used to distinguish *Entamoeba* isolates. Antibodies to the EhJacob2, EhJessie3, and chitinase each recognize cyst walls of clinical isolates of *Entamoeba*. While numerous sera from patients with amebic intestinal infections and liver abscess recognize recombinant EhJacob1 and EhJessie3 lectins, few of these sera recognize recombinant EhJacob2.

**Conclusions/Significance:**

The EhJacob2 lectin binds chitin and is polymorphic, and Jacob2, Jessie3, and chitinase are present in cyst walls of clinical isolates of *Entamoeba*. These results suggest there are substantial similarities between cysts of the human pathogen (Eh) and the *in vitro* model (Ei), even though there are quantitative and qualitative differences in their chitin-binding lectins.

## Author Summary

For many years, we and others have used cysts of *Entamoeba invadens* (Ei), a reptilian parasite, to model the infectious and diagnostic cysts of the human pathogen *Entamoeba histolytica* (Eh). The Ei cyst wall is composed of chitin fibrils, as well as Jacob and Jessie lectins that have unique chitin-binding domains. Our recent results suggest a “wattle and daub” model of the Ei cyst wall, where the wattle or sticks (chitin fibrils bound by multivalent Jacob lectins) is constructed prior to the addition of the mortar or daub (self-aggregating Jessie3 lectins). Here we “humanize” the Ei model of the cyst wall with four findings. First, a recombinant Eh Jacob2 lectin, which has three predicted chitin-binding domains surrounding a large spacer domain, binds chitin beads. Second, polymorphisms in the spacer domain of EhJacob2 discriminate clinical isolates of *Entamoeba*. Third, chitinase, Jacob2 lectin, and Jessie3 lectin are present in cyst walls of clinical isolates of *Entamoeba*. Finally, numerous sera from patients infected with *Entamoeba* recognize recombinant Eh Jacob1 and Jessie3 lectins.

## Introduction

The infectious and diagnostic stage of *Entamoeba histolytica* (Eh), the cause of amebic dysentery and liver abscess, is a quadranucleate cyst [1], [2]. Eh is morphologically indistinguishable from *Entamoeba dispar* (Ed), a human commensal that does not cause disease [3]. Because Eh does not readily encyst in axenic culture, we have studied cyst walls formed *in vitro* by *Entamoeba invadens* (Ei) that infects reptiles [4], [5].

The Ei cyst wall is composed of chitin (a homopolymer of β-1,4-linked GlcNAc) and three unique sets of chitin-binding lectins called Jacob, Jessie, and chitinase [6], [7]. Ei Jacob lectins contain 3 to 6 tandemly arranged chitin-binding domains (CBDs), each of which contains six Cys residues (see Table S1 for a list of database accession numbers and a brief description of each protein). Spacer regions between CBDs of Ei Jacob lectins contain sites for cleavage by Cys proteases, as well as Ser residues that are modified by O-phosphodiester-linked glycans [7]. Ei Jessie lectins and chitinase each contain an N-terminal CBD, which contains eight Cys residues [8]–[10]. Ei Jessie3 lectins contain a self-aggregating domain that forms the mortar or daub between chitin fibrils [11].

As Eh cysts are difficult to obtain from patient stool in quantity, we have predicted components of the cyst wall from the whole genome sequence of Eh [2], [12]. An Eh Jacob lectin (EhJacob1) that has two CBDs binds chitin when expressed as a recombinant protein in transfected Eh trophozoites (Table S1) [10]. Similarly, the N-terminal CBDs of Eh chitinase, Jessie2, and Jessie3 each bind chitin [10]. The Eh chitinase, chitin synthase, and chitin deacetylases each have the expected activities when expressed as recombinant proteins in bacteria or yeast [8], [13], [14]. Messenger RNAs for chitinases, Jessie lectins, and Jacob lectins are expressed by Eh encysting in xenic culture [15].

A low complexity spacer region between the CBD and enzymatic domain of Eh and Ed chitinases contains a series of heptapeptide repeats that are polymorphic among clinical isolates [16], [17]. Polymorphic tandem repeats have also been observed in the Ser-rich Eh protein (SREHP or K2 antigen) [16]–[19]. While abundant and immunogenic Eh trophozoite proteins such as the Gal/GalNAc lectin and SREHP are immunogenic and are therefore vaccine candidates [20]–[24], little is known about the immunogenicity of Eh cyst wall proteins.

In an effort here to test how well the Ei cyst model fits the human pathogen Eh, we characterized here a second Eh Jacob lectin (EhJacob2: EHI_044500; see Table S1) that contains three predicted CBDs separated by a long, Ser-rich spacer similar to those present in EiJacob6 and EiJacob7) [7]. Questions asked included the following:

- Does the EhJacob2 lectin bind chitin?
- Is the low complexity spacer of EhJacob2 polymorphic from isolate to isolate?
- Are Jacob2, Jessie3, and chitinase present in cyst walls of clinical isolates of *Entamoeba*?
- Do human anti-amebic sera recognize recombinant Eh cyst wall proteins?

## Materials and Methods

### Ethics statement

Culture and manipulation of *Entamoeba*, including production of cysts *in vitro* and handling of cysts from patient samples, has been has been approved by the Boston University Institutional Biosafety Committee (BU IBC). Similarly, recombinant expression of *Entamoeba* proteins in bacteria has been approved by the BU IBC. Rabbit antibodies were made using approved protocols from the BU IACUC. An exemption has been received from the Boston University IRB for de-identified patient sera and for de-identified stool samples containing *Entamoeba* cysts. Patient sera, all of which bound to Gal/GalNAc lectin, came from five individuals with amebic liver abscess and five individuals with intestinal amebiasis. All of these serum samples, which were de-identified, were collected prior to the initiation of these studies. The Ethical Review Committee of the International Centre for Diarrhoeal Disease Research, Bangladesh (ICDDR,B) and the Human Investigation Committee of the University of Virginia reviewed and approved the design of the previous study under which these samples were obtained.

### Identification of Eh and Ed Jacob2 lectins

Eh and Ed Jacob2 lectins were identified in BLASTP searches of the NR database at NCBI or at AmoebaDB using the EhJacob1 sequence (see Table S1 for accession numbers) [10], [12], [25]. N-terminal signals and transmembrane helices were predicted using Phobius [26].

### Analysis of Jacob2 gene polymorphisms

Genomic DNA from axenic Eh strains (HM-1:IMSS, HK-9, 200:NIH, and SD157) was isolated using the Wizard Genomic DNA Purification Kit (Promega). DNA from an axenic strain of Ed (SAW760) was a generous gift from Graham Clark. DNAs from numerous clinical isolates of Eh were a generous gift from Egbert Tannich. PCR primers flanking the Ser-rich region between the second and third CBDs of Jacob 2 were designed from sequences that were identical in the Eh and Ed genomic sequences. The sense primer (GCTGATGGATTCTACTGTGTT) encoded the heptapeptide (ADGFYCV). The anti-sense primer (ACAGAAAAGACCATCTTGAGT) was anti-sense to heptapeptide (TQDGLFC). In the Eh genome project strain HM-1:IMSS the predicted product was 1260-nt long [12]. PCR was performed for 35 cycles of 30 sec at 94°C, 30 sec at 50°C, and 3 min at 72°C using the PCR Master Mix system (Promega). Amplified products were analyzed using a 0.8% agarose gel in 1× Tris-acetate-EDTA (TAE) buffer. Selected PCR products were cloned into a TA-vector and sequenced from both ends.

### Expression of EhJacob2 in transfected amebae

The entire coding region of the EhJacob2 gene (1722 nt encoding a 574-aa protein) was PCR amplified from HM-1:IMSS strain gDNA using the Expand High Fidelity PCR system (Roche). The sense primer (GCGGTACC *ATG*AAACAACTTATATTAGCA) began at the start codon (italic) and included a *Kpn*I site (underline). The anti-sense primer (GCGGATCC *TTA* **TAAATCTTCTTCTGAAATTAATTTTTGTTCCTTGTTTTCATTGTTATTATT** ) included a *Bam*HI site (single underline) and was anti-sense to the 3′ end of the coding region of EhJacob2 (bold underline). This primer was anti-sense to a c-myc sequence (bold) and to a stop codon (italic). This product was cloned into the pJST4 vector [27] between the 5′ and 3′ untranslated regions of the Eh actin gene, and this construct was used to transfect HM-1:IMSS trophozoites. Transfected Eh trophozoites were lysed by incubation in lysis/wash buffer (20 mM Tris-HCl, pH 8.0; 1 M NaCl, 0.1% Triton X-100) plus 250 µM E64 for 1 hr on ice. The lysate was centrifuged for 1 min at maximum speed in a microcentrifuge, and the supernatant was incubated with chitin beads (New England Biolabs) for 1 hr at room temperature. Unbound material was then removed, and the beads were washed 5 times in lysis/wash buffer. Bound material was removed by boiling the beads for 5 min in SDS buffer (50 mM Tris-HCl, pH 6.8; 2% SDS; 5% 2-mercaptoethanol, 5% glycerol).

Protein samples were analyzed by SDS-PAGE on 4–20% Tris-glycine gels. After electrophoresis, proteins were stained with Coomassie Blue or blotted onto nitrocellulose. EhJacob2 was detected on the blots using an anti-c-myc antibody (Invitrogen) followed by a peroxidase-conjugated goat anti-mouse IgG antibody (Jackson ImmunoResearch). Bound antibodies were detected with the LumiGLO chemiluminescent substrate (KPL).

### Expression of Eh cyst wall proteins in bacteria and production of rabbit antibodies

The region of the EhJacob1 gene encoding a 53-aa C-terminal 6-Cys CBD, which begins with VNCTEVKE and ends with the stop codon, was PCR amplified from Eh DNA. The sense primer (CGGGATCCGTCAATTGTACTGAAGTGAAAGAA) had a *Bam*HI site at the 5′-end (underline). The anti-sense primer (CCCAAGCTT *TTA* **GTGGTGGTGGTGGTGGTGATAACATGGATTGTTATAAC** ), which had a 5′ *Hind*III site (underlined), was anti-sense to a stop codon (italics), a polyHis tail (bold), and the 3′ end of the coding region of the EhJacob1 gene (bold underline).

The coding region of the EhJacob2 gene (minus the first 48 nt that encode the N-terminal 16-aa-long signal peptide and minus 24-aa at the C-terminus) was PCR amplified from Eh DNA. The sense primer (GGGTACCTAATGGTATACCCAACTGGATGTAAGAAGAAA) had a *Kpn*1 site at the 5′-end (underline) and encoded the peptide (VYPTGCKKK) that is C-terminal to the predicted cleavage site in EhJacob2 for the signal peptidase [26]. The anti-sense primer ( GGATCC *TTA* **GTGGTGGTGGTGGTGGTGGTATTGGTAAGGACCTTCTTGT** ), which had a *Bam*H1 site (underline), was anti-sense to a stop codon (italics), a polyHis tail (bold), and the 3′ end of the coding region of the EhJacob2 gene (bold underline). The EhJacob1 and EhJacob2 PCR products were cloned into pMAL-p2E (New England Biolabs), using the same methods we used to clone Eh Jessie3 into this vector [11]. Maltose-binding protein (MBP) fusions containing the Eh cyst wall lectins were expressed in *E. coli* (Bl21-DE3 strain) using IPTG induction and an amylose resin (NEB) for purification. The purity of these recombinant proteins was checked on SDS-PAGE.

The 363-aa long catalytic domain of Eichitinase1, which begins with the peptide (KVVSYYT) was amplified from Ei DNA using a sense primer ( GGATCCATGAAGGTTGTCTCGTATTACACC) that had a 5′ *Kpn*I site (underline). The anti-sense primer ( CTCGAG *TTA* **GCAACCGATCAAGCTCTTTC** ) had a *Xho*I site (underline) and was anti-sense to a stop codon (italic) and the C-terminal peptide (KKELDQC) (bold underline). The Eichitinase1 PCR product was cloned into the pQE30 vector (Qiagen) and expressed in M15 strain of *E. coli* that contains a lac repressor-expressing plasmid (pREP4). Recombinant Eichitinase1, which contains a C-terminal polyHis tag, was induced with IPTG and purified on Ni-NTA agarose beads.

Mono-specific polyclonal rabbit antibodies to amylose resin-purified MBP-EhJacob1 and MBP-EhJacob2 were made at Strategic Biosolutions, using methods similar to that used previously to make a rabbit anti-EhJessie3 antibody [11]. Prior to their use in microscopy, rabbit antibodies were purified using MBP-EhJacob1 or MBP-EhJacob2 fusion-proteins chemically coupled to agarose. Similar methods were used to raise a mono-specific rabbit antibody to the catalytic domain of Eichitinase1. This antibody cross-reacts with the catalytic domain of Ehchitinase1.

### Binding of anti-cyst wall lectin antibodies to *Entamoeba* cysts in stool samples

Approximately 2 to 3 grams of stool sample from patients infected with *Entamoeba* (in Kharagpur, India) were emulsified in 10 ml of chilled phosphate-buffered saline (PBS) and then passed through a mesh to remove the larger particles from the materials. Each sample was washed with ice cold PBS by centrifugation at 5000 rpm for 5 min thrice, and the pellet was resuspended in 1 ml PBS. The presence of *Entamoeba* cyst in sample was confirmed by iodine staining and light microscopy or by calcofluor white staining and epifluorescent microscopy.

To localize the Jacob2, chitinase and Jessie3 in the Eh cyst wall, we fixed stool samples fixed with 2% paraformaldehyde for 15 min at room temperature and washed three times with PBS. Fixed samples were incubated for two hrs with 1∶200 dilutions of rabbit anti-EhJacob2, anti-Eh Jessie3, and anti-chitinase (catalytic domain) antibodies (described above). Samples were washed with PBS and then incubated with TRITC-conjugated goat anti-rabbit antibody (1∶500 dilution) for 1 hr. Secondary antibody alone was used as negative control. Samples were again washed with PBS and examined with a FV1000 confocal microscope (Olympus). Images were captured by FV10-ASW 1.6 viewer and processed with Adobe photoshop CS3.

Finally, xenic cysts of Eh, which were incubated with anti-Jacob2 and anti-Jessie3 antibodies, were a generous gift of Upinder Singh [15].

### Binding of anti-amebic sera to Western blots of recombinant cyst wall lectins

For Western blotting, ∼2 µg each of MBP, MBP-EhJacob1, MBP-EhJacob2 and MBP-EhJessie3 were separated in 4–16% gradient SDS-PAGE (Invitrogen, USA) and transferred to a PVDF membrane by semi-dry method. Blotted membranes were incubated with each patient's sera (1∶10 dilution) (Dacca, Bangladesh) on a rocker overnight at 4°C. Membranes were washed three times for 15 min with PBS-Tween 20 and then incubated with HRP-conjugated anti-human antibody (Sigma) (1∶2000 dilution) for 1 hr. Bound antibody was detected using Super Signal West Pico Chemiluminescent kit (Pierce), as per manufacturer's instruction. The strength of bound antibodies was qualitatively scored as no signal (−), barely detectable signal (+/−), weak signal (+), stronger signal (++), and strongest signal (+++).

## Results and Discussion

### The EhJacob2 lectin has a large Ser-rich spacer

**Figure 1:**
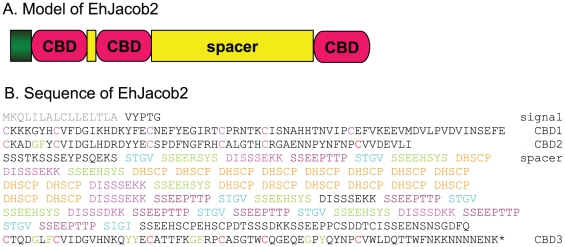
Eh Jacob2 has three chitin-binding domains (CBDs) surrounding a large Ser-rich spacer. A. EhJacob2 has an N-terminal signal peptide, three CBDs, and a large spacer between the first two CBDs and the last CBD. EhJacob2 has no transmembrane helix or GPI-anchor. B. Sequence of EhJacob2 where signal peptide (grey) and Cys residues (red) within CBDs are highlighted. Also highlighted are short repeats in the spacer, which fall into five families: A (light blue), B (green), C (pink), D (purple), and E (orange). Polymorphisms in these repeat families are further described in Fig. 4. The Ed Jacob2 is shown in Fig. S1.

Eh has only two predicted Jacob lectins [2], [10], [12]. EhJacob1, which we previously characterized [10], is present in three nearly identical copies in the genome (see Table S1). EhJacob1, which contains two CBDs, is 151-amino acids long, has a predicted molecular weight of 17377 daltons, and has a predicted pI of 5.2. In contrast, the EhJacob2 lectin, which contains three predicted CBDs, is 574-amino acids long, has a predicted molecular weight of 62862 daltons, and has a predicted pI of 4.65 (Figs. 1A and 1B). The first two predicted CBDs of EhJacob2 are separated from the third CBD by a large spacer domain, which is 30% Ser. Large Ser-rich spacer domains are also present in minor components of the Ei cyst wall (EiJacob6 and EiJacob7) and in chitin-binding proteins of insects (peritrophins) that are present in the wall surrounding the blood meal [7], [28]. Large Ser-rich domains in EhJacob2 suggest the possibility of numerous O-phosphodiester-linked glycans, as demonstrated in Ei Jacob lectins [7]. In contrast, there are no sites for Asn-linked glycosylation in EhJacob2 [29]. Within the spacer domain of EhJacob2 are numerous short repeats that are polymorphic (see next section). These repeats include sequences (e.g. TTPSTGV) that resemble sites for cleavage by Cys proteases in Ei Jacob lectins (TTPVD) [7].

The predicted Jacob2 from the commensal parasite Ed (EDI_246160) is 743-amino acids long and contains three CBDs that closely resemble those of EhJacob2 (Fig. S1 and Table S1). In contrast, the Ser-rich domain of EdJacob2 contains numerous repeats (marked in bold letters in Fig. S1) that are distinct from those of EhJacob2.

### The EhJacob2 lectin binds chitin

**Figure 2:**
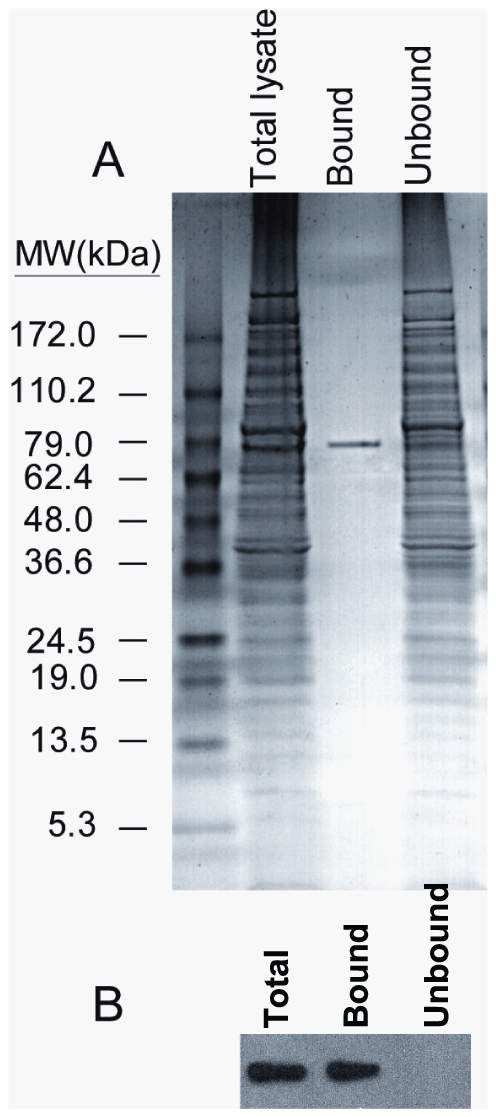
EhJacob2 is a chitin-binding protein. A. Coomassie blue-stained SDS-PAGE gel showing a total lysate of Eh trophozoites transfected with c-myc tagged EhJacob2, the fraction that binds chitin beads, and the fraction that does not bind chitin. B. Western blot confirms the chitin binding of EhJacob2, which is detected with anti-c-myc antibodies and chemiluminescence.

To determine if EhJacob2 is a chitin-binding lectin, EhJacob2 was expressed with a myc-tag at its C-terminus in transfected Eh trophozoites [10], [27]. A total lysate from transfected Eh was incubated with chitin beads, and unbound proteins (the vast majority) were removed (Fig. 2A). A single, ∼78-kDa protein binds to the chitin beads. A Western blot using an anti-myc antibody confirmed that this chitin-binding protein is the recombinant EhJacob2 protein (Fig. 2B). In control non-transfected *E. histolytica* trophozoites, no proteins bind to chitin beads (data not shown).

### The Ser-rich domain of EhJacob2 is highly polymorphic

**Figure 3:**
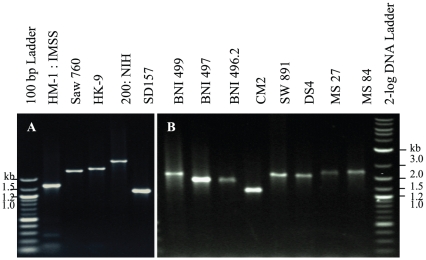
Ser-rich domains of EhJacob2 are polymorphic. Amplification products were generated using PCR primers flanking the Ser-rich region between the second and third chitin-binding domains of Jacob2. A. Jacob2 PCR products from axenized Eh isolates (HM-1:IMSS, HK-9, 200:NIH, and SD157) and Ed isolate (SAW760) have distinct mobilities on agarose gels. B. Jacob2 PCR products from clinical Eh isolates also have distinct mobilities.

We hypothesized that the Ser-rich region of EhJacob2 might be polymorphic because similar low complexity regions containing internal repeats in *Entamoeba* SREHP and chitinase are polymorphic [16]–[19]. EhJacob2 PCR products from DNA of axenized strains of Eh (HM-1:IMSS, HK-9, and 200:NIH), one clinical isolate (SD157), and axenized Ed strain (SAW760) range in size from 1.1 kb to 2.3 kb (Fig. 3A). EhJacob2 PCR products also range in size from clinical isolates of Eh (Fig. 3B).

**Figure 4:**
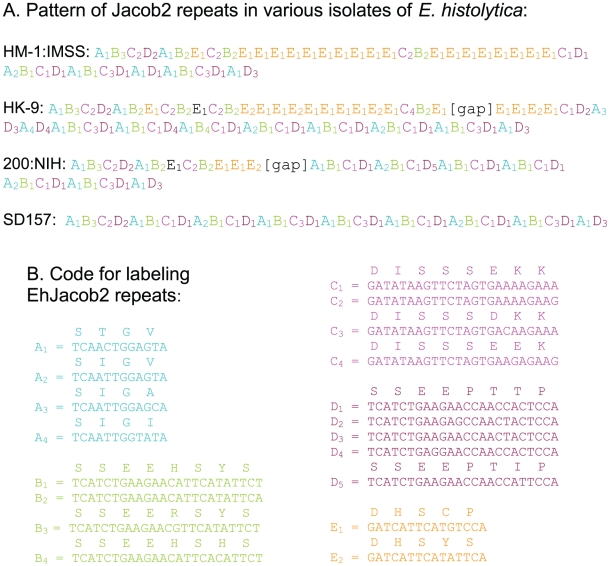
EhJacob2 PCR products are distinct for each axenized strain. A. Coded representations of EhJacob2 repeats from PCR products shown in Fig. 3. Complete sequences were obtained for HM-1:IMSS and SD157. Gaps in the middle of sequences in the HK-9 and 200:NIH products are marked. B. Five EhJacob2 repeats are each assigned a letter (A to E) and a color (as described in Fig. 1). The nucleotide sequences coding for each repeat are numbered in the order of their frequency of occurrence in the sequenced products.

Selected EhJacob2 PCR products were cloned and sequenced at both ends, and five groups of repetitive elements in the Ser-rich spacer were coded (A to E in Fig. 4), using methods to describe *Entamoeba* chitinase and Ser-rich protein repeats [16]. Nucleotide differences within groups included both silent and non-silent changes. While the repetitive elements differ among the four isolates examined, there are some similarities. For example, the repetitive regions all start with A_1_B_3_C_2_D_2_A_1_ and end with D_1_A_1_B_1_C_4_D_1_A_1_D_3_. Blocks of ABCD are common, and HM-1:IMSS, HK-9, and 200:NIH all have CB followed by variable numbers of E. In contrast, E repeats did not occur in the SD157 sequence.

#### Antibodies to the EhJacob2, EhJessie3, and chitinase each recognize cyst walls of clinical isolates of *Entamoeba*

**Figure 5:**
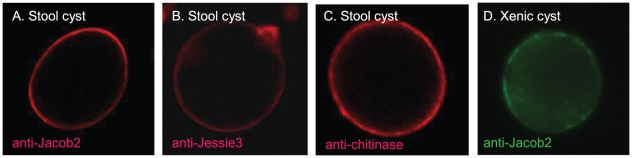
Antibodies to EhJacob2, Jessie3, and chitinase bind to *Entamoeba* cysts isolated from patient stools. A to C. Confocal micrographs of stool cysts detected with rabbit antibodies to Eh Jacob2, Jessie3, or chitinase. D. Confocal micrograph of an Eh cyst from xenic culture labeled with antibodies to EhJessie3. Thanks to Upinder Singh for the micrograph shown in D.

Previously we made polyclonal, mono-specific rabbit antibodies to recombinant EiJacob1 and EhJessie3 and to a heptapeptide repeat present in the spacer domain of the Ei chitinase [11]. Sequential binding of these antibodies to cysts of Ei was used to develop the “wattle and daub” model of Ei cyst wall formation [11]. Here we made polyclonal, mono-specific rabbit antibodies to the entire EhJacob2 and the catalytic domain of Ei chitinase (that is nearly identical to catalytic domains of Eh and Ed chitinases). Antibodies to EhJacob2, EhJessie3, and the catalytic domain of *Entamoeba* chitinases do not bind to Eh trophozoites but bind to cysts of *Entamoeba* isolated from patient stools (Fig. 5). Because these cysts were not characterized by molecular methods, we do not know whether they are composed of Eh, Ed, or both. Because the Eh and Ed sequences for Jacob2, Jessie3, and chitinases are so similar (Table S1), we assume but have not proven that antibodies to these cyst wall proteins react with cysts of both Eh and Ed. In addition, anti-EhJacob2 antibodies but not anti-EhJessie3 antibodies bind to Eh cysts made in xenic culture [15].

### Anti-amebic sera recognize to varying degrees recombinant EhJacob1, EhJacob2, and EhJessie3 lectins

The idea here was to determine whether sera from patients with liver abscess or amebic intestinal infection, each of which recognizes the Gal/GalNAc lectin of Eh trophozoites [22], [23], also recognize recombinant Eh cyst wall proteins on Western blots. MBP alone was used as negative control. While 9 of 10 human anti-amebic sera recognized EhJessie3, 6 of 10 sera recognized EhJacob1 (Table 1). In contrast, just 2 of 10 sera bound to EhJacob2, suggesting EhJacob2 may be less antigenic than the other Eh cyst wall lectins.

**Table 1:** Binding of human anti-amebic sera to recombinant Eh cyst wall lectins.

Sl.	Sera Number	Jessie 3*	Jacob1	Jacob2
1	LAI 09	+	+++	−−
2	LAI 12	+	+	−−
3	LAI 28	++	−−	−−
4	LAI 30	+	+++	+
5	LAI 43	++	+	−−
6	041	+	+	+
7	163	+	−−	−−
8	1022	+/−	−−	−−
9	1028	+	++	−−
10	3040	−−	−−	−−

*The strength of bound antibodies was qualitatively scored as no signal (−), barely detectable signal (+/−), weak signal (+), stronger signal (++), and strongest signal (+++).

### Major conclusions and unresolved questions

The results here and elsewhere generally support the idea that Ei is a good model for Eh cysts:

- Eh Jacob lectins have a similar structure to those described for Ei, and both bind chitin when expressed as recombinant proteins (Figs. 1 and 2) [10]. As an aside, EhJacob2 shows the best expression of any protein we have tried to overexpress in transfected trophozoites. Whether Eh Jacob lectins have post-translational addition of O-phosphodiester-linked glycans to Ser in the spacer domains and cleavage by endogenous Cys proteases, as shown for Ei [7], cannot be determined using the present experimental strategy. Whether differences in the repetitive elements of EhJacob2 and EdJacob2 (Fig. S1) can be exploited for diagnostic purposes also remains to be determined.
- The major components of the Ei cyst wall (Jacob lectins, Jessie lectins, and chitinase), all of which contain unique CBDs, are also present in *Entamoeba* cyst walls of clinical isolates (Fig. 5) [6]–[11]. The finding that Eh cysts from xenic cultures bind anti-Jacob antibodies but not anti-Jessie3 antibodies suggests the possibility that the *in vitro* cysts may have an incompletely assembled wall [15]. This is because in the Ei model, Jacob lectins are added to cyst walls prior to addition of Jessie lectins [11].
- Eh Jacob lectins, Jessie3 lectins, and chitinase are immunogenic in rabbits [6], [11], and it appears that EhJessie3 and EhJacob1 are immunogenic in some persons infected with *Entamoeba* (Table 1). Whether the immune response to *Entamoeba* cyst wall lectins inhibits encystation or excystation and so has an effect on transmission of cysts from person to person is interesting but cannot be determined from this data. In contrast, a mucosal IgA anti-lectin antibody response is associated with immune protection against Eh colonization in Bangladeshi children [22], [23].
- Differences between Eh and Ed cysts and cysts of Ei include the failure of Eh or Ed to encyst in axenic culture using the conditions that cause Ei to encyst [4], [15]. Ei has seven distinct Jacob lectin genes rather than two present in Eh and Ed (Table S1) [2], [7], [10], [12]. Eh and Ed each has a single chitinase with a C-terminal CBD, while Ei has three chitinases with an N-terminal CBD and two chitinases that have no CBD [8], [9]. Eh and Ed each has a single Jessie3 lectin, while Ei has two Jessie3 lectins [7], [10].

Finally, it appears that EhJacob2 genes are at least as polymorphic as SREHP genes and are more polymorphic than chitinase genes [16]–[19]. These results support the general idea that polymorphisms in surface proteins that contain repetitive elements of *Entamoeba*, *Cryptosporidium* (e.g. gp40/15), and *Plasmodium* (e.g. merozoite and circumsporozoite antigens) may be used to distinguish clinical isolates [30]–[32]. The EhJacob2 polymorphisms may complement other methods such as tRNA gene-linked tandem repeats for finger-printing clinical isolates of Eh [33], [34].

## Supporting Information

Figure S1 Ed Jacob2 differs from Eh Jacob2 primarily in the large Ser-rich spacer. Sequence of EdJacob2 (Table S1) where the signal peptide (grey) and Cys residues (red) within CBDs are highlighted (see Fig. 1 for comparison to EhJacob2). Also highlighted are short repeats in the spacer, which fall into five families: A (light blue), B (green), C (pink), D (purple), and E (orange). Differences between the sequence of EdJacob2 and EhJacob2 are marked in bold letters. Because the number and arrangement of these short repeats differs between EdJacob2 and EhJacob2, it was not possible to directly align the two sequences.
(2.68 MB EPS)

Table S1 *Entamoeba* proteins with chitin-binding domains (CBDs).
(0.05 MB DOC)
