# Supplementary material for: The Jacob2 Lectin of the Entamoeba histolytica Cyst Wall Binds Chitin and Is Polymorphic
Source: PLoS Negl Trop Dis. 2010 Jul 20;4(7):e750. doi: 10.1371/journal.pntd.0000750 (PMC2907411; doi:10.1371/journal.pntd.0000750)
Supplement: Table S1 — Entamoeba proteins with chitin-binding domains (CBDs). (0.05 MB DOC) [file pntd.0000750.s002.doc]

Table S1. *Entamoeba* proteins with chitin-binding domains (CBDs).

Protein AmoebaDB length 6-cys 8-Cys other features Experiments

EhJacob1 [EHI_028930](http://amoebadb.org/amoeba/showRecord.do?name=GeneRecordClasses.GeneRecordClass&project_id=AmoebaDB&primary_key=EHI_028930)^1^ 151-aa 2 0 [10,here]

EdJacob1 [EDI_136500](http://amoebadb.org/amoeba/showRecord.do?name=GeneRecordClasses.GeneRecordClass&project_id=AmoebaDB&primary_key=EDI_136500) (85%)

EhJacob2 [EHI_044500](http://amoebadb.org/amoeba/showRecord.do?name=GeneRecordClasses.GeneRecordClass&project_id=AmoebaDB&primary_key=EHI_044500) 574-aa 3 0 very large spacer here

EdJacob2 [EDI_246160](http://amoebadb.org/amoeba/showRecord.do?name=GeneRecordClasses.GeneRecordClass&project_id=AmoebaDB&primary_key=EDI_246160) (92%)^2^

EiJacob1^3^ [EIN_050710](http://amoebadb.org/amoeba/showRecord.do?name=GeneRecordClasses.GeneRecordClass&project_id=AmoebaDB&primary_key=EIN_050710) 401-aa 5 0 [6,7]

EiJacob2 [EIN_137570](http://amoebadb.org/amoeba/showRecord.do?name=GeneRecordClasses.GeneRecordClass&project_id=AmoebaDB&primary_key=EIN_137570) 419-aa 5 0 [7]

EiJacob3 [EIN_016240](http://amoebadb.org/amoeba/showRecord.do?name=GeneRecordClasses.GeneRecordClass&project_id=AmoebaDB&primary_key=EIN_016240) 351-aa 5 0 [7]

EiJacob4 EIN_294450 AmoebaDB EIN_294450 399-aa 5 0 [7]

EiJacob5 [EIN_104770](http://amoebadb.org/amoeba/showRecord.do?name=GeneRecordClasses.GeneRecordClass&project_id=AmoebaDB&primary_key=EIN_104770) 204-aa 3 0 [7]

EiJacob6 [EIN_015880](http://amoebadb.org/amoeba/showRecord.do?name=GeneRecordClasses.GeneRecordClass&project_id=AmoebaDB&primary_key=EIN_015880) 1021-aa 6 0 very large spacer [7]

EiJacob7 [EIN_186850](http://amoebadb.org/amoeba/showRecord.do?name=GeneRecordClasses.GeneRecordClass&project_id=AmoebaDB&primary_key=EIN_186850) 681-aa 3 0 very large spacer [7]

Ehchitinase1 [EHI_109890](http://amoebadb.org/amoeba/showRecord.do?name=GeneRecordClasses.GeneRecordClass&project_id=AmoebaDB&primary_key=EHI_109890) 507-aa 0 1 catalytic domain [8,10,here]

Edchitinase1 [EDI_120190](http://amoebadb.org/amoeba/showRecord.do?name=GeneRecordClasses.GeneRecordClass&project_id=AmoebaDB&primary_key=EDI_120190) (95%)^4^

Eichitinase1 [EIN_084170](http://amoebadb.org/amoeba/showRecord.do?name=GeneRecordClasses.GeneRecordClass&project_id=AmoebaDB&primary_key=EIN_084170)

Eichitinase [EIN_289570](http://amoebadb.org/amoeba/showRecord.do?name=GeneRecordClasses.GeneRecordClass&project_id=AmoebaDB&primary_key=EIN_289570)

Eichitinase EIN_239240

Eichitinase [EIN_059870](http://amoebadb.org/amoeba/showRecord.do?name=GeneRecordClasses.GeneRecordClass&project_id=AmoebaDB&primary_key=EIN_059870) 381-aa 0 0 catalytic domain [9]

Eichitinase [EIN_053310](http://amoebadb.org/amoeba/showRecord.do?name=GeneRecordClasses.GeneRecordClass&project_id=AmoebaDB&primary_key=EIN_053310)

EhJessie3 [EHI_152170](http://amoebadb.org/amoeba/showRecord.do?name=GeneRecordClasses.GeneRecordClass&source_id=EHI_152170&project_id=AmoebaDB) 621-aa 0 1 daub domain [10,11,here]

EdJessie3 [EDI_038370](http://amoebadb.org/amoeba/showRecord.do?name=GeneRecordClasses.GeneRecordClass&project_id=AmoebaDB&primary_key=EDI_038370) (96%)

EiJessie3 [EIN_040990](http://amoebadb.org/amoeba/showRecord.do?name=GeneRecordClasses.GeneRecordClass&project_id=AmoebaDB&primary_key=EIN_040990) [7,10,11]

EiJessie3 [EIN_058620](http://amoebadb.org/amoeba/showRecord.do?name=GeneRecordClasses.GeneRecordClass&project_id=AmoebaDB&primary_key=EIN_058620)

EhJessie1 [EHI_024660](http://amoebadb.org/amoeba/showRecord.do?name=GeneRecordClasses.GeneRecordClass&project_id=AmoebaDB&primary_key=EHI_024660) 90-aa 0 1 [10]

EdJessie1^5^ predicted (97%)

EhJessie2 [EHI_092100](http://amoebadb.org/amoeba/showRecord.do?name=GeneRecordClasses.GeneRecordClass&project_id=AmoebaDB&primary_key=EHI_092100) [10]

EdJessie2^5^ predicted (84%)

EhJessie4 [EHI_180790](http://amoebadb.org/amoeba/showRecord.do?name=GeneRecordClasses.GeneRecordClass&project_id=AmoebaDB&primary_key=EHI_180790)

EdJessie4^5^ predicted (93%)

EiJessie1 EIN_059900

EiJessie1 EIN_312260

EiJessie1 EIN_243430

^1^EhJacob1 is present in three nearly identical copies: [EHI_028930](http://amoebadb.org/amoeba/showRecord.do?name=GeneRecordClasses.GeneRecordClass&project_id=AmoebaDB&primary_key=EHI_028930), EHI_067190, and EHI_136360.

^2^Identities are shown for chitin-binding domains but do not include Ser-rich spacer that is not alignable between EhJacob2 and EdJacob2. For differences in these regions see Fig. S1.

^3^EiJacob1 is present in two nearly identical copies: [EIN_050710](http://amoebadb.org/amoeba/showRecord.do?name=GeneRecordClasses.GeneRecordClass&project_id=AmoebaDB&primary_key=EIN_050710) and [EIN_051170](http://amoebadb.org/amoeba/showRecord.do?name=GeneRecordClasses.GeneRecordClass&project_id=AmoebaDB&primary_key=EIN_051170).

^4^Identities are shown for chitin-binding domains and catalytic domains but do not include Ser-rich spacer that is not alignable between Ehchitinase1 and Edchitinase1. See Refs [16,17] for characterization of these spacers.

^5^EdJessie1, EdJessie2, and EdJessie4 were each identified by TBLASTN of Ed genomic sequences. Why these proteins were absent from the set of Ed genes is not clear.
